# Impact of Different Sampling Schemes for Decision Making in Soil-Transmitted Helminthiasis Control Programs

**DOI:** 10.1093/infdis/jiz535

**Published:** 2019-12-12

**Authors:** Luc E Coffeng, Veronica Malizia, Carolin Vegvari, Piet Cools, Katherine E Halliday, Bruno Levecke, Zeleke Mekonnen, Paul M Gichuki, Somphou Sayasone, Rajiv Sarkar, Ame Shaali, Johnny Vlaminck, Roy M Anderson, Sake J de Vlas

**Affiliations:** 1 Department of Public Health, Erasmus MC, University Medical Center Rotterdam, Rotterdam, The Netherlands; 2 London Centre for Neglected Tropical Disease Research, Department of Infectious Disease Epidemiology, Imperial College London, London, United Kingdom; 3 Department of Virology, Parasitology and Immunology, Faculty of Veterinary Medicine, Ghent University, Belgium; 4 Faculty of Infectious and Tropical Diseases, London School of Hygiene & Tropical Medicine, London, United Kingdom; 5 Jimma University Institute of Health, Jimma University, Jimma, Ethiopia; 6 Eastern and Southern Africa Centre of International Parasite Control, Kenya Medical Research Institute, Nairobi, Kenya; 7 Lao Tropical and Public Health Institute, Ministry of Health, Vientiane, Lao People’s Democratic Republic; 8 Division of Gastrointestinal Sciences, Christian Medical College, Vellore, Tamil Nadu, India; 9 Laboratory Division, Public Health Laboratory-Ivo de Carneri, Chake Chake, United Republic of Tanzania

**Keywords:** fecal egg counts, Kato-Katz, moderate-to-heavy infection, prevalence, soil-transmitted helminths

## Abstract

Starting and stopping preventive chemotherapy (PC) for soil-transmitted helminthiasis is typically based on the prevalence of infection as measured by Kato-Katz (KK) fecal smears. Kato-Katz-based egg counts can vary highly over repeated stool samples and smears. Consequentially, the sensitivity of KK-based surveys depends on the number of stool samples per person and the number of smears per sample. Given finite resources, collecting multiple samples and/or smears means screening fewer individuals, thereby lowering the statistical precision of prevalence estimates. Using population-level data from various epidemiological settings, we assessed the performance of different sampling schemes executed within the confines of the same budget. We recommend the use of single-slide KK for determining prevalence of moderate-to-heavy intensity infection and policy decisions for starting and continuing PC; more sensitive sampling schemes may be required for policy decisions involving stopping PC. Our findings highlight that guidelines should include specific guidance on sampling schemes.

Soil-transmitted helminthiases (STHs), caused by *Ascaris lumbricoides*, *Trichuris trichiura*, and hookworms, affect approximately 1.5 billion people worldwide. The World Health Organization (WHO) has targeted STH for control as a public health problem by 2020 through regular preventive chemotherapy (PC), with control defined as prevalence of moderate-to-heavy (MH) intensity infection ≤1% [1–3]. The WHO further recommends that decisions on starting, continuing, and stopping PC are based on estimates of STH prevalence (any intensity) in school-age children as measured by the classic parasitological Kato-Katz (KK) fecal smear [1–3].

Kato-Katz-based egg counts vary highly within individuals over repeated stools samples from different days and between repeated slides based on the same stool sample. Consequently, surveys based on multiple stool samples per individual and/or multiple slides per stool sample typically yield higher prevalence estimates than when examining 1 slide per person [4]. However, increasing the number of slides per sample or the number of stool samples per person (requiring visiting a study site multiple days) means fewer individuals can be tested for the same amount of resources, thereby lowering the statistical precision of prevalence estimates. The trade-off between sensitivity and precision of survey results will be different for prevalence of any versus MH intensity infection, because examining multiple slides increases sensitivity but does not affect estimates of mean intensity of infection [4]. The WHO currently provides a blanket recommendation that prevalence of STH should be evaluated based on a single stool sample per child [2], without any specific recommendation for the number of slides that should be prepared per sample.

In this study, we explore how different sampling schemes used within the confines of the same budget affect the sensitivity of surveys to detect infection (any and MH intensity) and the statistical precision of surveys results. We do so by bootstrap analysis of existing datasets from different epidemiological settings where multiple stool samples were collected and/or stool samples were examined multiple times.

## METHODS

### Datasets

Four datasets covering 6 countries were analyzed; Appendix A provides an overview of characteristics of each dataset, including geographical location, sample size, dominant STH species, and history of control. The first dataset constituted information from a population-based survey conducted in 2008 in a subcounty of eastern Uganda [5]. Members of all households in the subcounty were asked to provide 2 stool samples on 2 consecutive days (26.3% of participants provided only 1 sample); each sample was examined with duplicate KK. The second dataset held baseline data from an epidemiological study in 45 villages in southern India [6], including up to 3 stool samples per individual (10.8% and 6.4% of individuals returned only 1 or 2 stools samples, respectively), each sample examined once with the McMaster egg counting technique (which yields qualitatively similar results to KK [7]) if initial screening with saline and iodine wet preparation was positive. The third dataset constituted baseline data from the TUMIKIA study, a cluster-randomized trial in 120 clusters of villages in Kenya [8, 9], in which 1 individual per household was tested with duplicate KK based on a single stool sample. The fourth dataset contained baseline data from the Starworms study [10–12] in which school-age children in Ethiopia, Lao People’s Democratic Republic, and Tanzania were tested using (among other techniques) duplicate KK performed on a single stool sample. The Starworms study was the only study in which stool samples were homogenized before preparing the fecal smears. Hookworm was the dominant STH species in all sites, except for the Starworms study sites, where roundworm (*A lumbricoides*) and whipworm (*T trichiura*) were equally prevalent.

Datasets were prepared in such a way that all individuals within a single dataset had the same number of records by explicitly including incomplete or missing records as missing values, to account for the reality that stool sample collection, slide preparation, and/or slide examination may be incomplete or fail.

### Data Analysis

For each dataset, we defined a set of sampling schemes according to which the survey might have been (alternatively) conducted within the confines of the available data. We defined sampling schemes as a×b, where a is the number of stool samples per person, and b is the number of repeated slides per sample. Expected differences in prevalences (absolute and relative, a×b=1×1 as reference) resulting from different sampling schemes were quantified based on 10 000 repeated bootstraps [13]. Within each bootstrap iteration, we started by sampling a number of individuals (with replacement) equal to the original size of the dataset. Then, for each individual, we randomly shuffled stools samples (if more than 1 was available, keeping together repeated slides based on the same sample), and for each stool sample, we randomly shuffled repeated slides (if any). For each sampling scheme a×b, we saved the bootstrapped egg counts from the a first stool samples and b first slides per sample for each individual in the bootstrapped data. This way, within each bootstrap iteration, the first examined slide of each individual included in a multislide sampling scheme was also included in the 1×1 reference scheme, allowing us to directly compare results of the different sampling schemes as if executed on the same set of candidate people.

Results of each sampling scheme were summarized in terms of prevalence of any infection and prevalence of MH intensity infection. Individuals were considered positive if at least 1 of their slides was positive for eggs. Individuals were considered positive for MH intensity infection based on the number of eggs per gram feces (epg) averaged over all slides, using cutoffs for MH intensity infection as defined by WHO [2]: ≥2000 epg for hookworm species, ≥1000 epg for *T trichiura*, and ≥5000 epg for *A lumbricoides*. Individuals with all egg counts missing were discarded from the bootstrapped data before calculations, without replacement.

To compare sensitivity and statistical precision of different sampling schemes when executed within the confines of the same budget, we performed a second bootstrap procedure. First, we defined a budget B (range, 50 up to 2000), where the value of Bis the number of individuals that can be tested within a 1×1 sampling scheme. Then, for the other schemes, we calculated the number of people that could be tested with the same budget as follows: $N_{a\times b}=B/\left(S_{a\times b}\times C_{b}\times a\right)$. Here, $S_{a\times b}$ is the total number of slides per person in scheme a×b (eg, 4 for a 2×2 scheme), and $C_{b}$ is the relative cost per slide for single ($C_{1}=1$) or duplicate slides ($C_{2}=\frac{cost\;per\;slide_{1\times 2}}{cost\;per\;slide_{1\times 1}}=\frac{2.41/2}{1.94}=0.621$, based on published cost estimates [14]). The last term a in the denominator is included as a multiplier assuming that collecting a samples per person will increase cost per slide a-fold due to having to visit the study site a days. For instance, given a budget worth the cost of testing 500 individuals in a 1×1 scheme (B=500), the number of individuals that can be tested in a 1×2 scheme ($S_{a\times b}=2$, $C_{b}=0.621$, a=1) would be $N_{1\times 2}=500/\left(2\times 0.621\times 1\right)=402$, rounded down to the nearest integer. Likewise, for a 2×1 scheme ($S_{a\times b}=2$, $C_{b}=1$, a=2), the number of individuals that can be tested would be $N_{2\times 1}=500/\left(2\times 1\times 2\right)=125$, and for a 2×2 scheme ($S_{a\times b}=4$, $C_{b}=0.621$, a=2) the number of individuals would be $N_{2\times 2}=500/\left(4\times 0.621\times 2\right)=100$.

For each value of budget B, we performed 10 000 repeated bootstrap iterations. Each bootstrap iteration started by sampling B individuals (with replacement) for the 1×1 scheme and shuffling stools samples and repeated slides (if any) as before. Subsequently, for each non-1×1 sampling scheme, we selected the $N_{a\times b}$ first people from the bootstrapped dataset, and we selected the first a stool samples per individual and first b slides per sample. This way, again, all individuals included in the non-1×1 schemes (and their first examined slide) were also included in the 1×1 reference scheme. Prevalences were calculated and compared as in the first bootstrap analysis. Results of the budget-constrained bootstrap analysis were summarized by means of boxplots (illustrating the variation in prevalence of any and/or MH infections), and the proportion of bootstrap iterations in which the estimated prevalence from a non-1×1 scheme was higher than the corresponding estimate from the 1×1 reference scheme.

## RESULTS

Table 1 provides the added value of performing a second slide from the same stool sample. In the Ugandan and Kenyan datasets, this significantly increased the estimated prevalence by a factor of 1.25 for low-endemic settings (prevalence ≤5%) and 1.10 in highly endemic settings (prevalence ≥45%), whereas absolute difference in prevalence were highest for more highly endemic settings (see Table 1 for details). For the Starworms data, testing a second slide did not increase estimated prevalences, which was due to the reduced variation between repeated slides resulting from stool homogenization [15]. This is also reflected by the relatively low number of discordant (zero/nonzero) pairs of duplicate egg counts (eg, 0.8% of pairs in Tanzania hookworm data vs 9.0% in the Ugandan data). This pattern was similar for all 3 STH species and in all 3 countries covered by the Starworms study. Appendix B summarizes the added value of collecting additional stool samples per person, which increased prevalences by up to a factor of 1.59 (2×1 scheme) and 1.98 (3×1 scheme). Again, absolute increases in prevalence were highest in more highly endemic settings (see Appendix B for details). In the Indian data, the 3×1 scheme increased prevalence to a lesser extent (1.25 relative to 2×1 scheme) compared with the 2×1 scheme (1.59 relative to 1×1 scheme). In the Ugandan dataset, the increase in prevalence was higher in the 2×1 scheme (1.45) than in the 1×2 scheme (1.19).

**Table 1.** Differences in Estimates of Prevalence of Hookworm Infection (Any Intensity) Based On Single (1×1) or Duplicate Slide (1×2) Examination of a Single Stool Sample^a^

Dataset	Expected Results for 1×1 Scheme		Expected Difference in Prevalence Between 1×2 and 1×1 Schemes	
	Prevalence of Infection (95% CI)	Mean Egg per Gram (95% CI)	Absolute (95% CI)	Relative (95% CI)
Mulanda, Tororo, Uganda	23.4 (21.3–25.4)	258 (181–350)	4.5 (3.3–5.5)	1.19 (1.15–1.24)
Kwale, Kenya (TUMIKIA)	16.7 (16.1–17.2)	168 (149–191)	2.4 (2.2–2.6)	1.14 (1.13–1.16)
Prevalence ≤5%	2.4 (1.8–2.9)	11 (5–17)	0.6 (0.3–0.9)	1.25 (1.13–1.40)
Prevalence 5%–15%	8.8 (8.1–9.5)	81 (62–105)	1.5 (1.2–1.8)	1.17 (1.14–1.21)
Prevalence 15%–25%	17.2 (16.1–18.3)	167 (135–200)	2.6 (2.1–3.1)	1.15 (1.12–1.18)
Prevalence 25%–35%	26.1 (24.6–27.7)	299 (220–417)	3.9 (3.2–4.7)	1.15 (1.12–1.18)
Prevalence 35%–45%	35.5 (33.1–37.8)	381 (295–480)	4.4 (3.3–5.5)	1.12 (1.09–1.16)
Prevalence >45%	48.1 (45.2–51.2)	465 (381–556)	5.0 (3.6–6.4)	1.10 (1.07–1.14)

Abbreviations: CI, confidence interval.

^a^Estimates are based on 10 000 bootstraps of the entire datasets. For the Kenyan data, data were also stratified based on overall prevalence at the cluster level (N = 120). Results for the Starworms data are not shown because differences between the 2 sampling schemes were nonsignificant for all of the worm species in all of the countries. Absolute differences are expressed as percentage points; relative differences are expressed as ratios of 1×2 over 1×1. The 95% CI is based on the 2.5th and 97.5th percentiles of bootstrap results.

Prevalence of MH intensity infection did not differ significantly between any of the sampling schemes for any of the datasets, because the 95% confidence intervals for relative differences always included the value 1.0. Only in the Indian data did estimates tend to decline with additional stool samples (again nonsignificantly), which could be explained by the 2-tiered testing method in which eggs were only counted if initial screening of the sample was positive. The resulting introduction of zero counts (where eggs might have actually been found with the McMaster) caused mean egg counts for individuals to decline when more stool samples were included in the prevalence calculation.

**Figure 1.**
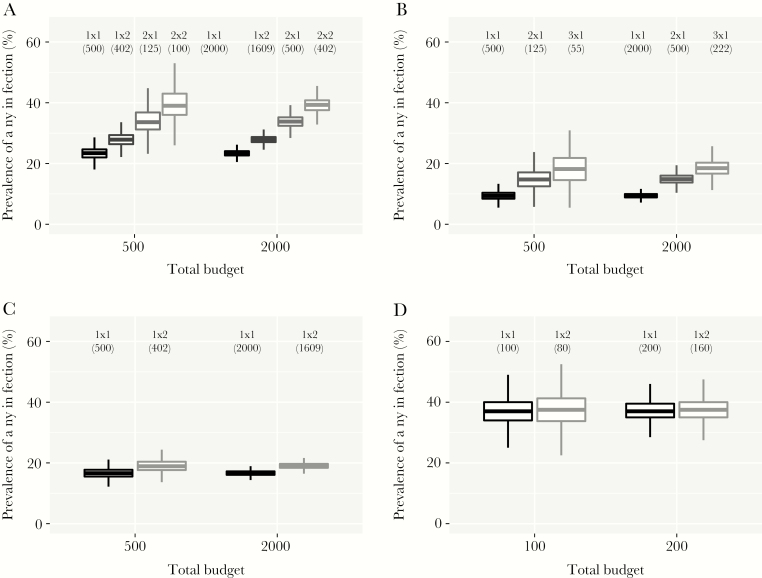
Sensitivity and precision of different sampling schemes for detection of hookworm infection, when based on the same overall budget. Budget is expressed as the number of individuals that can be tested in the context of a 1×1 scheme (1 stool sample collected per person and 1 slide tested per sample). Numbers between brackets under the sampling scheme indicators (“1×1”, “1×2”, etc) represent the number of people who were tested in that scheme, given the budget. Boxes represent the median and interquartile range (25th and 75th percentiles) of the bootstrapped prevalences; whiskers cover the range of bootstrapped values up to a distance of 1.5 times the interquartile range from the outer hinges of the box. Estimated are based on 10 000 bootstraps. (A) Ugandan dataset [5]; (B) Indian dataset [6]; (C) Kenyan data (TUMIKIA) [8, 9]; (D) Tanzanian dataset (Starworms) [10–12].

**Figure 2.**
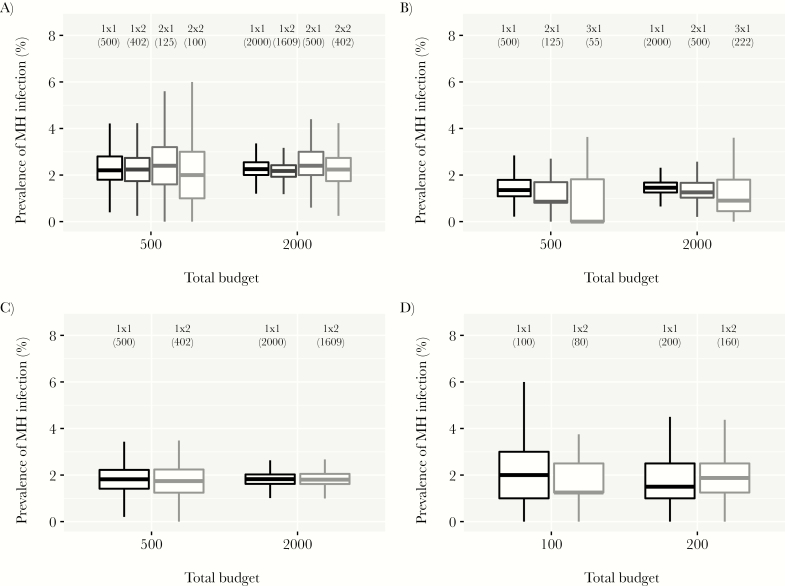
Sensitivity and uncertainty of different sampling schemes for detection of moderate-to-heavy intensity hookworm infection in the general population, based on the same overall budget. Budget is expressed as the number of individuals that can be tested in the context of a 1×1 scheme (1 stool sample collected per person and 1 slide tested per sample). Numbers between brackets under the sampling scheme indicators (“1×1”, “1×2”, etc) represent the number of people who were tested in that scheme, given the budget. Boxes represent the median and interquartile range (25th and 75th percentiles) of the bootstrapped prevalences; whiskers cover the range of bootstrapped values up to a distance of 1.5 times the interquartile range from the outer hinges of the box. Estimated are based on 10 000 bootstraps. (A) Ugandan dataset [5]; (B) Indian dataset [6]; (C) Kenyan data (TUMIKIA) [8, 9]; (D) Tanzanian dataset (Starworms) [10–12].

Figure 1 shows that when sampling schemes are restrained by the same budget, the reference 1×1 scheme produced survey prevalence estimates with the highest statistical precision, because it included the highest number of people. In general, non-1×1 schemes produced higher prevalence estimates, but with a risk of occasionally finding a lower prevalence than the 1×1 scheme. However, in the Starworms study sites, non-1×1 schemes only increased statistical uncertainty of prevalence estimates without a gain in sensitivity (Figure 1D; see Appendix C for all countries and worm species in the Starworms data). For all datasets but the Starworms data, the risk of accidentally finding a lower prevalence with a non-1×1 scheme decreased with higher budgets. The minimum budget to reduce this risk to ≤5% depended on endemicity (eg, a budget equivalent to the cost of testing 1030 and 122 individuals in a 1×1 scheme for the lowest and highest endemicity strata in the Kenyan data, Appendix D). In the endemicity-stratified analysis of the Kenyan data, the choice of scheme (1×1 vs 1×2) determined the probability that the estimated prevalence of any infection was under or above a policy-relevant threshold value (Appendix E). Figure 2 illustrates the impact of sampling scheme on prevalence of MH intensity infection: non-1×1 schemes only increased statistical uncertainty without a gain in sensitivity. Results were qualitatively similar for all species and sites in the Starworms data (Appendix F).

## DISCUSSION

We show that when designing surveys for assessing the epidemiological situation of STH within a finite budget, a trade-off exists between the sensitivity of the survey to detect infection and the statistical precision of survey results. Importantly, gains in sensitivity only apply to prevalence of infection (any intensity) based on unhomogenized stools and are most pronounced in low-endemic settings (on a relative scale). For prevalence of MH intensity infection, sampling multiple stool samples per person and/or slides per sample only increases statistical uncertainty and not sensitivity.

Because prevalence estimates depend on the choice of sampling scheme, it is important that threshold values for policy decision regarding starting, changing, or stopping PC are defined in conjunction with a clearly specified survey strategy, ie, with explicit mention of the numbers of samples per person and slides per sample tested. If deviating survey strategies are used, survey results should be translated to the standard scale, using the relative differences (and associated uncertainty) estimated here and elsewhere [4]. Based on our analyses, we recommend that policy decisions for starting and continuing PC are based on a 1×1 sampling scheme, because survey results will then be most stable, ensuring the most consistent policy. However, when deciding whether to stop PC (threshold prevalence of any infection of 2% [2]), sensitivity of the sampling scheme will be particularly important because false-negative findings may lead to stopping PC prematurely. In such situations the higher costs of multiple sampling probably outweigh the costs of potentially having to restart the control program. Schemes based on multiple stool samples per person will be of particular value if a survey using the 1×1 scheme (executed within the confines of the same budget) would require spending multiple days in a site (eg, for large populations), because this would partially negate the additional costs of having to spend multiple days to collect multiple stool samples per person. We further expect that for low-endemic settings, relative gains in sensitivity when using multiple stool samples per person will be higher than estimated here, because prevalence will typically be lower than in the data used here (lowest prevalence of infection approximately 9% in Indian data), and because relative gains in sensitivity increase with declining prevalence (Kenyan data).

A major strength of our analysis and improvement over previous analyses [4] is that we include cost considerations when comparing sampling schemes, while at the same time accounting for the reality that not all individuals return (all) samples and that slides preparation and examination may fail. Context-dependent costs of sampling schemes [16] can be easily accounted for by multiplying the budget value in our analysis with the cost per slide in a 1×1 scheme in that particular context. Furthermore, context-dependent cost differences between sampling schemes can be easily included by adapting 2 parameters for cost per sample and cost per slide. However, to predict the outcomes of sampling schemes for epidemiological situations far outside the data, such as situations with very low infection levels after a prolonged period of PC, will require either of 2 approaches. The first is the collection of data from those contexts and analyzing them in the same fashion as done here. The ongoing Deworm3 study [17] is expected to generate such data (including a 1×2 KK scheme), in the context of intense school-based and community-based PC for STH after an initial period of community-wide PC for lymphatic filariasis. The second approach is to use transmission models to predict the distribution of infection intensities over time during PC and combine these with a statistical model for the distribution of egg counts in stool samples and slides [15]. This is the subject of ongoing work for both STH and schistosomiasis within the NTD Modelling Consortium, which focuses on the development of transmission models that realistically account for heterogeneity in exposure to transmission and uptake of PC in populations to make policy-relevant predictions of the impact of control efforts [18–24].

Homogenization results in a more Poisson-like distribution of repeated egg counts [15], which has lower variation in contrast to the typically overdispersed, negative binomial distribution of repeated egg counts [25, 26]. This means that for individuals with low or high egg loads (which typically constitute the majority of endemic populations due to overdispersed worm loads), test sensitivity will be maximized with 1 slide per sample, which matches our finding of no increase in sensitivity for multiple-slide KK based on homogenized stools. Repeated slides based on homogenized stools are therefore unlikely to noticeably increase survey sensitivity, but sampling stool samples over multiple days will still increase survey sensitivity. Theoretically, stool homogenization increases the sensitivity of surveys to detect infection, with the highest absolute increase in individuals with moderate to high egg densities [27]. Whether the sensitivity of a single slide based on homogenized stool is higher than that of multiple slides based on unhomogenized stool depends on the overall egg density in a stool sample and therefore the distribution of egg densities in the population. These complexities warrant a more detailed evaluation of the performance of different sampling strategies in context of control programs, which again requires a combination of transmission models and statistical models.

As for the appropriateness of the WHO-recommended thresholds for STH policy, these cannot be evaluated by this study, because their appropriateness depends on the following: (1) whether they adequately reflect to what extent STHs pose a significant public health problem, and (2) whether starting, changing, or stopping PC around the current threshold values actually results in achieving the target of <1% prevalence MH intensity infection. The first aspect needs to be addressed by quantifying the association between infection levels and morbidity and their dynamics during control. The second aspect has been partly addressed by a recent modeling study, which suggested that current thresholds for scaling down PC frequency are inappropriate because infection levels will likely bounce back to levels above the current morbidity target [28]. A follow-up modeling study further demonstrated how the positive predictive value of the prevalence of infection (any intensity) in a sample of sentinel villages for having reached the morbidity target in a wider geographical area highly depends on precontrol endemicity, the dominant STH species, number of sentinel villages sampled, and variation in infection levels within the wider geographical area [29].

## CONCLUSIONS

In conclusion, we show that different sampling strategies lead to considerable variation in the policies recommended under current WHO guidelines, highlighting the need to link thresholds for decision making to specific sampling schemes. When policy decisions are based on KK, we recommend the use of single slides, unless the decision involves stopping PC, in which case multisample KK is better, in particular in sites where a single-slide survey of the population requires multiple days. Prevalence of MH intensity infection should always be evaluated using a single slide per person.

## Supplementary Data

Supplementary materials are available at *The Journal of Infectious Diseases* online. Consisting of data provided by the authors to benefit the reader, the posted materials are not copyedited and are the sole responsibility of the authors, so questions or comments should be addressed to the corresponding author.

jiz535_suppl_Supplementary_Appendix_A

jiz535_suppl_Supplementary_Appendix_B

jiz535_suppl_Supplementary_Appendix_C

jiz535_suppl_Supplementary_Appendix_D

jiz535_suppl_Supplementary_Appendix_E

jiz535_suppl_Supplementary_Appendix_F

## Notes

***Acknowledgments.*** We acknowledge several people who contributed to the collection of the data that were kindly shared with them for use in the current study: William Oswald, Simon Brooker, Charles Mwandawiro, Carlos Mcharo, and Rachel Pullan for sharing the TUMIKIA dataset from Kenya; Rachel Pullan and Simon Brooker for sharing the Ugandan dataset; Gagandeep Kang for sharing the Indian dataset; and Mio Ayana and Daniel Dana for sharing the Starworms dataset from Ethiopia.

***Author contributions.*** L. E. C. contributed to concept and design. R. S., K. E. H., Z. M., S. A., S. S., P. C., and J. V. contributed to data collection. V. M. and L. E. C. contributed to data analysis. All authors contributed to data interpretation. L. E. C., V. M., and C. V. contributed to drafting of manuscript. All authors contributed to critical review and revision of manuscript.

***Disclaimer.*** The funders had no role in study design, data collection and analysis, decision to publish, or preparation of the manuscript. The views, opinions, assumptions or any other information set out in this article are solely those of the authors and should not be attributed to the funders or any person connected with the funders.

***Financial support.*** L. E. C., V. M., C. V., R. M. A., and S. J. d. V. gratefully acknowledge funding of the NTD Modelling Consortium by the Bill & Melinda Gates Foundation (OPP1184344). L. E. C. further acknowledges funding from the Dutch Research Council (NWO, Grant 016.Veni.178.023). R. S. acknowledges funding from Wellcome Trust/DBT India Alliance (Grant number IA/E/12/1/500750). The Starworms study was supported by a grant from the Bill & Melinda Gates Foundation (OPP1120972, principal investigator is B. L., www.starworms.org). P. C. was financially supported by a grant from the Bill & Melinda Gates Foundation (OPP1120972, www.starworms.org). B. L. was a postdoctoral fellow of the Research Foundation (www.FWO.be). J. V. was financially supported through an International Coordination Action of the Flemish Research Foundation.

***Supplement sponsorship.*** The authors acknowledge funding of the NTD Modelling Consortium by the Bill and Melinda Gates Foundation (OPP1184344).

***Potential conflicts of interest.*** All authors: No reported conflicts of interest. All authors have submitted the ICMJE Form for Disclosure of Potential Conflicts of Interest.
