# Supplementary material for: Impact of Different Sampling Schemes for Decision Making in Soil-Transmitted Helminthiasis Control Programs
Source: J Infect Dis. 2019 Dec 12;221(Suppl 5):S531–8. doi: 10.1093/infdis/jiz535 (PMC7289558; doi:10.1093/infdis/jiz535)
Supplement: jiz535_suppl_Supplementary_Appendix_A [file jiz535_suppl_supplementary_appendix_a.pdf]

Appendix A. Overview of datasets and study sites used in the analysis. Only baseline data were used from each of the datasets. All Kato-Katz (KK) faecal smears were based on 41.7 mg of stool.

Location	Study design	Sample size				Age groups sampled	Dominant species	History of control	Parasitological technique	Prevalence of infection (%)		Reference
		Individuals	Households	Villages	Clusters of villages					Any	Moderate-to-heavy intensity	
Mulanda, Tororo, Uganda	Household-based census	2,037	469	4	1	6 months and older	Hookworm	Ongoing control[a]	2 repeated KK[b] on a single sample per day for 2 consecutive days (2x2)	39.2	2.4	[5]
Jawadhu Hills, Tamil Nadu, India	Community-based cluster-randomized trial	2,082	824	45	1	2 to 70 years	Hookworm	Ongoing control[c]	Single McMaster[d] on a single sample per day for up to 3 consecutive days (3x1)	18.5	1.0	[6]
Kwale, Kenya (TUMIKIA)	Community-based cluster-randomized trial	19,684	19,674	940	120	2 years and older	Hookworm	Ongoing control[e]	2 repeated KK[b] on a single stool sample (1x2)	19.1	1.7	[8,9]
Nam Bak, Laos People's Democratic Republic (Starworms)	School-based drug efficacy trial	239	-	-	3 schools	5 to 14 years	Hookworm, whipworm, roundworm	Ongoing control[f]	2 repeated KK[g] on a single stool sample (1x2)	90	25.1	[10–12]
Pemba Island,Tanzania (Starworms)	School-based drug efficacy trial	245	-	-	4 schools	5 to 14 years	Hookworm, whipworm, roundworm	Ongoing control[f]	2 repeated KK[g] on a single stool sample (1x2)	37	2.0	[10–12]
Jimma, Ethiopia (Starworms)	School-based drug efficacy trial	161	-	-	2 schools	5 to 14 years	Hookworm, whipworm, roundworm	Ongoing control[f]	2 repeated KK[g] on a single stool sample (1x2)	41.6	1.2	[10–12]

[a] Three rounds of school-based deworming at 30%-65% coverage during four years preceding the study.
[b] Kato-Katz faecal smear based on 41.7 mg of stool, without homogenisation of individual stool samples.
[c] Annual mass drug adminsitration with diethylcarbamazine and albendazole since 2007 for control of lymphatic filariasis, with the annual treatment round typically taking place in July. The state-level treatment coverage was reported as 97.3% (data specific to the study area was not available); however, given the hilly terrain of the study area the actual coverage was expected to be much lower. The baseline data used from this study was collected over a period of one year, between October 2013 and November 2014.
[d] McMaster was based on 2 grams of stool, as described elsewhere [7], but only on samples in which eggs where detected by saline/iodine wet slide first; samples that scored negative on the saline/iodine wet slide were scores contained zero eggs.
[e] The area was subject to four rounds of community-based mass drug administration with albendazole and diethylcarbamazine for lymphatic filariasis (2003, 2005, 2008, and 2011, coverage levels of 62.7% and 58.3% in the last two rounds). The area further received four rounds of school-based deworming with albendazol for STH between, with programmatic coverage of 82% of pre-school and school-age children (2-14 years) in 2014. The baseline data used from this study was collected between March and May 2015.
[f] Based on the reported national coverage of drug administration to pre-school-age and school-age children for the last 5 years (2009-2014; Preventive Chemotherapy Database of the World Health Organization), the site in Ethiopia was considered to have experienced a low drug exposure (treatment with albendazole since 2015), the site in Lao PDR a medium drug exposure (treatment with mebendazole since 2007), and the site in Pemba (Tanzania) a high drug exposure (treatment with albendazole since 1994) prior to the start of the study.
[g] Kato-Katz faecal smear based on 41.7 mg of stool; e individual stool sample was homogenised with a wooden spatula before slide preparation.
