# Supplementary material for: Impact of Different Sampling Schemes for Decision Making in Soil-Transmitted Helminthiasis Control Programs
Source: J Infect Dis. 2019 Dec 12;221(Suppl 5):S531–8. doi: 10.1093/infdis/jiz535 (PMC7289558; doi:10.1093/infdis/jiz535)
Supplement: jiz535_suppl_Supplementary_Appendix_B [file jiz535_suppl_supplementary_appendix_b.pdf]

Appendix B: Differences in estimates of prevalence of hookworm infection (any intensity) based on single (1x1) or multiple stool samples, examined by means of single (2x1, 3x1) or duplicate slides (2x2). Estimated are based on 10,000 bootstraps of the entire datasets. Absolute differences are expressed as percentage-points; relative differences are expressed as factors. 95%-CI = 95% confidence interval based on the 2.5th and 97.5th percentiles of bootstrap results.

Dataset	Expected results for 1x1 scheme		Average absolute difference in prevalence compared to the 1x1 scheme (95%-CI)			Average relative difference in prevalence compared to the 1x1 scheme (95%-CI)		
	Prevalence of infection (%) [a]	Mean egg count (epg)	2x1	2x2	3x1	2x1	2x2	3x1
Mulanda, Tororo, Uganda	23.4 (21.3–25.4)	258 (181–350)	10.4 (8.9–12.1)	15.8 (14.1–17.7)	-	1.45 (1.36–1.55)	1.68 (1.57–1.80)	-
Jawadhu Hills, Tamil Nadu, India	9.4 (7.8–11.1)	126 (87–181)	5.4 (4.1–6.9)	-	9.1 (7.5–10.5)	1.59 (1.40–1.82)	-	1.98 (1.72–2.3)
