# Supplementary material for: Impact of Different Sampling Schemes for Decision Making in Soil-Transmitted Helminthiasis Control Programs
Source: J Infect Dis. 2019 Dec 12;221(Suppl 5):S531–8. doi: 10.1093/infdis/jiz535 (PMC7289558; doi:10.1093/infdis/jiz535)

Appendix C: Sensitivity and uncertainty of different sampling schemes of all three soil-transmitted helminth species in school-age children in the Starworms dataset, based on the same budget. Budget is expressed as the number of individuals that can be tested in the context of a 1x1 scheme (one stool sample collected per person and one slide tested per sample). Boxes represent the median and interquartile range (25th and 75th percentiles) of the bootstrapped prevalences; whiskers cover the range of bootstrapped values up to a distance of 1.5 times the interquartile range from the outer hingers of the box. The left (darker) box for each budget value represents the 1x1 sampling scheme, the right (lighter) box represents the 1x2 sampling scheme. Estimated are based on 10,000 bootstraps.

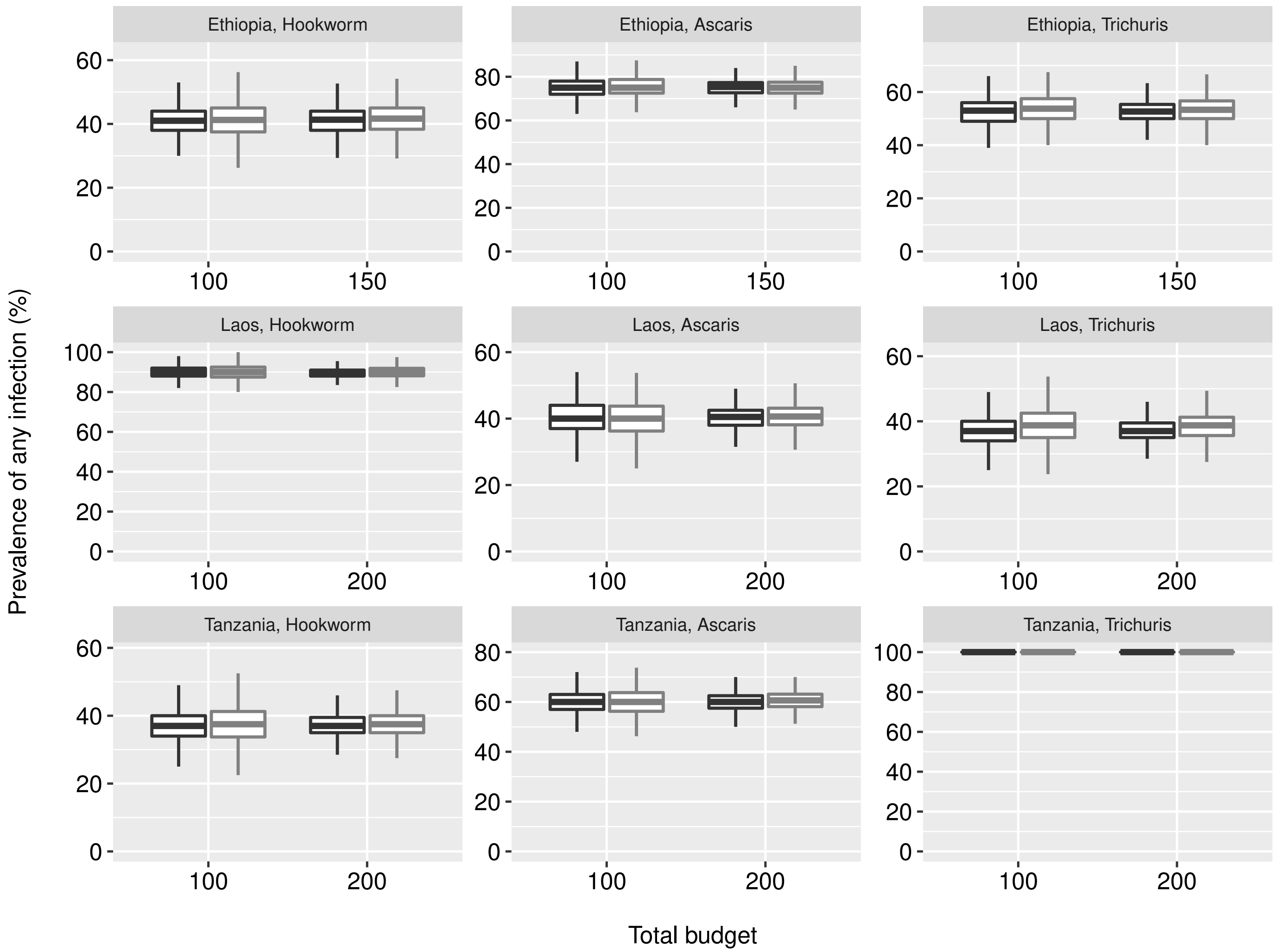

Supplement: jiz535_suppl_Supplementary_Appendix_C [file jiz535_suppl_supplementary_appendix_c.pdf]
