# Supplementary material for: Impact of Different Sampling Schemes for Decision Making in Soil-Transmitted Helminthiasis Control Programs
Source: J Infect Dis. 2019 Dec 12;221(Suppl 5):S531–8. doi: 10.1093/infdis/jiz535 (PMC7289558; doi:10.1093/infdis/jiz535)
Supplement: jiz535_suppl_Supplementary_Appendix_D [file jiz535_suppl_supplementary_appendix_d.pdf]

Appendix D. Minimum number of individuals to sample with a non-1x1 scheme for a ≤5% risk of accidentally finding a lower prevalence than in a 1x1 scheme based on the same budget. Associated budget values are indicated in brackets behind minimum number of individuals. Budget values are expressed in terms of the number of individuals that can be sampled within a 1x1 sampling scheme for the same budget. Results for the Starworms data are not shown as non-1x1 sampling schemes did no significantly improve sensitivity for detection of any of the worm species in any of the countries.

Dataset	Expected results for 1x1 scheme		Minimum number of individuals required to better detect infection with scheme with 95% certainty (budget)			
	Prevalence of infection	Mean egg per gram	1x2	2x1	2x2	3x1
Mulanda, Tororo, Uganda	23.4	258	-	48 (192)	24 (120)	-
Jawadhu Hills, Tamil Nadu, India	9.4	126	-	99 (396)	-	48 (432)
Mulanda, Tororo, Uganda	23.4	258	96 (120)	-	-	-
Kwale, Kenya (TUMIKIA)	16.7	168	235 (292)	-	-	-
Prevalence ≤5%	2.4	11	829 (1,030)	-	-	-
Prevalence 5-15%	8.8	81	359 (446)	-	-	-
Prevalence 15-25%	17.2	167	206 (256)	-	-	-
Prevalence 25-35%	26.1	299	122 (152)	-	-	-
Prevalence 35-45%	35.5	381	122 (152)	-	-	-
Prevalence >45%	48.1	465	98 (122)	-	-	-
